# The magnitude and perceived reasons for childhood cancer treatment abandonment in Ethiopia: from health care providers’ perspective

**DOI:** 10.1186/s12913-022-08188-8

**Published:** 2022-08-08

**Authors:** Mizan Kiros Mirutse, Mieraf Taddesse Tolla, Solomon Tessema Memirie, Michael Tekle Palm, Daniel Hailu, Kunuz Abdella Abdi, Ermias Dessie Buli, Ole F. Norheim

**Affiliations:** 1 Ministry of Health of Ethiopia, Addis Ababa, Ethiopia; 2 Department of Global Public Health and Primary Care, Bergen Centre for Ethics and Priority Setting (BCEPS), University of Bergen, Bergen, Norway; 3 Addis Center for Ethics and Priority Setting, College of Health Sciences, Addis Ababa University, Addis Ababa, Ethiopia; 4 Clinton Health Access Initiative, Addis Ababa, Ethiopia; 5 Department of Pediatrics and Child Health, Pediatric Hematology/Oncology Unit, College of Health Sciences, Addis Ababa University, Addis Ababa, Ethiopia; 6 World Health Organization, Addis Ababa, Ethiopia

**Keywords:** Childhood cancer, Treatment abandonment, Low-income countries, Sub-Saharan Africa, Ethiopia

## Abstract

**Background:**

Treatment abandonment is one of major reasons for childhood cancer treatment failure and low survival rate in low- and middle-income countries. Ethiopia plans to reduce abandonment rate by 60% (2019–2023), but baseline data and information about the contextual risk factors that influence treatment abandonment are scarce.

**Methods:**

This cross-sectional study was conducted from September 5 to 22, 2021, on the three major pediatric oncology centers in Ethiopia. Data on the incidence and reasons for treatment abandonment were obtained from healthcare professionals. We were unable to obtain data about the patients’ or guardians’ perspective because the information available in the cancer registry was incomplete to contact adequate number of respondents. We used a validated, semi-structured questionnaire developed by the International Society of Pediatric Oncology Abandonment Technical Working Group. We included all (*N* = 38) health care professionals (physicians, nurses, and social workers) working at these centers who had more than one year of experience in childhood cancer service provision (a universal sampling and 100% response rate).

**Results:**

The perceived mean abandonment rate in Ethiopia is 34% (SE 2.5%). The risk of treatment abandonment is dependent on the type of cancer (high for bone sarcoma and brain tumor), the phase of treatment and treatment outcome. The highest risk is during maintenance and treatment failure or relapse for acute lymphoblastic leukemia, and during pre- or post-surgical phase for Wilms tumor and bone sarcoma. The major influencing risk factors in Ethiopia includes high cost of care, low economic status, long travel time to treatment centers, long waiting time, belief in the incurability of cancer and poor public awareness about childhood cancer.

**Conclusions:**

The perceived abandonment rate in Ethiopia is high, and the risk of abandonment varies according to the type of cancer, phase of treatment or treatment outcome. Therefore, mitigation strategies to reduce the abandonment rate should include identifying specific risk factors and prioritizing strategies based on their level of influence, effectiveness, feasibility, and affordability.

**Supplementary information:**

The online version contains supplementary material available at 10.1186/s12913-022-08188-8.

## Background

Globally, close to 400,000 new cases of childhood (age range, 0–19 years) cancer are reported annually [1], and low- and middle-income countries (LMICs) account for a large proportion (90%) of these cases [2–4]. The prognosis of childhood cancer varies from complete cure to near-certain death, depending on which part of the world the child is born in. The 5-year survival rate in high-income countries (HICs) is more than 80% [5–9], while it is 20% to 30% in LMICs [4, 10, 11] and can be as low as 10% in some East African Countries [12]. This drastic difference in survival can be explained by poor availability of and access to specialized pediatric oncology treatment centers and supportive care, diagnostic centers, drugs and other treatment supplies, trained human resources, social support, late presentation, and high treatment abandonment rate in LMICs [10, 13].

Treatment abandonment is one of the major factors for treatment failure and low survival rate in LMICs [14–20]. The International Society of Pediatric Oncology defines treatment abandonment as failure to start (refusal) or continue treatment for four or more consecutive weeks [16]. This does not include those with medical contraindications for the treatment or those who are transferred to other centers or lost to follow up after completion of treatment. A systematic literature review conducted in 2007 included nearly 50 studies conducted between 1992 and 2006 that examined the incidence of abandonment of childhood cancer treatment in LMICs [15]. The study showed abandonment of treatment was associated with all the major childhood cancer types and was found to be an issue across the LMICs. There was a high degree of variation in the incidence rate of abandonment reported by the reviewed studies: it fell within the 10%–25% range in most studies, but some studies reported as high as 50%–60% [15].

There have also been studies targeting health professionals as the source of understanding magnitude and reasons for abandonment. For example, a global study conducted in 2012 utilized online surveys to interview 602 health professionals from 101 countries distributed across all income categories, including 10 low-income countries (LICs) and 26 LMICs [14]. The study found large disparities in the magnitude of abandonment between HICs and LMICs: 91% of HICs reported a median abandonment rate of less than 5%, while only 37% of LMICs did so. This study included eight Sub-Saharan African LICs and LMICs, among which three reported a median abandonment rate of 6%–15%; two reported a median abandonment rate of 26%–50%; and three reported a median abandonment rate of > 50% [14]. Importantly, Ethiopia was one of the three countries that reported a median abandonment rate of more than 50% [14]. Further, studies conducted in Kenya found that 50%–54% of children diagnosed with malignant cancers abandoned treatment [21–24], and the corresponding abandonment rate was 45% in Zambia [25], 42% in Ghana [26], 35% in Sudan [27], 33% in Uganda [28], and 19% in Malawi [29].

A systematic review and metanalysis (published in 2017) conducted in Sub-Saharan Africa [30], as well as other country-specific studies, have found that the common reasons for treatment abandonment are supply-side barriers such as high cost of care (associated with therapy, diagnostics, food, and lodging), lack of insurance, long travel time, long waiting time, and lack of social support, and demand-side barriers such as low income, cost of transport, poor public awareness, and fear [14, 21, 23, 24, 31, 32]. Abandonment of treatment is observed as a major problem even in settings where treatment is provided for free. For example, in Zambia, where free healthcare is provided, data for the period 2008–2010 indicated a high treatment abandonment rate, 45% [25]. A study conducted in a Malawi hospital that also provides free treatment explored the common reasons for abandonment, apart from treatment fee [33]. The study found that even though the patients’ families did not have to pay for treatment, they were deterred by other costs related to the treatment of their child, such as the cost of transport to and from the facility (which is a direct cost), as well as indirect costs such as the opportunity cost of labor income lost while being away from home. Another study on malignancies in patients below 16 years conducted between 2001 and 2003 in El Salvador found that low income and large household size were predictors of treatment abandonment [34], even when the cost of treatment was taken care of by relevant organizations. In addition to these factors, in Sub-Saharan African countries, preference for complementary and alternative medicine, strong faith and religious beliefs, competing priorities, and the notion that childhood cancer is an incurable illness highly influence abandonment [14].

According to the 2019–2023 Federal Ministry of Health of Ethiopia (FMoH)’s National Childhood and Adolescent Cancer Control Plan (NCACCP), addressing abandonment is one of the priority strategic objectives for improving the survival rate of children and adolescents with cancer [35]. The FMoH’s plan is to reduce abandonment by 60% over the 5-year period of 2019 to 2023 [35], but there is a lack of baseline data. In addition, the intervention areas to be prioritized and addressed have not been clearly identified in the NCACCP because there is not enough contextualized evidence. Therefore, the rationale behind our research is to generate contextualized data on the magnitude of and reasons for abandonment of childhood cancer treatment in Ethiopia. We believe that our study will serve as a baseline to monitor progress over time and will shed light on the major contextual risk factors associated with treatment abandonment. Thus, our research could augment policy making and the implementation of mitigation strategies to improve abandonment in Ethiopia and other closer settings.

## Methods

### Study setting

This study was conducted in Ethiopia—a country with a population of more than 100 million [36]. Ethiopia has a three-tier health delivery system that is composed of close to 300 public hospitals (including general and tertiary hospitals), 21,000 primary care public facilities (including health posts, health centers, and primary hospitals), 40,000 community health extension workers, 7,000 private clinics, and 70 private hospitals [37]. Ethiopia has made remarkable progress in the provision of primary health care, but access to specialty care, including pediatric oncology services, is poor. For example, there is a very limited number of pediatric oncology treatment and diagnostic centers, pediatric hematologist-oncologists (only seven as of 2021), oncology nurses, and pathologists [35]. The first pediatric oncology unit, located in Tikur Anbessa Specialized Hospital (TASH), was established in 2013, and recently, three additional centers, namely, Jimma University Hospital (JUH), Mekelle University Hospital (MUH), and Gondar University Hospital (GUH) have been added [35].

### Study design and sampling

We used a cross-sectional study design and sampled three out of the four pediatric oncology centers (TASH, JUH, and GUH) in the country. The original design considered all four centers, but the pediatric oncology center in MUH was excluded at a later stage for security reasons. We interviewed all health care professionals (physicians, nurses, and social workers) working at these centers who had more than one year of experience in childhood cancer care service provision. The inclusion criteria were physicians, nurses, and social workers that have direct engagement on the management of a child with cancer. The exclusion criteria was less than one year of experience in childhood cancer care.

We had originally planned to include the perspectives of both the patients’ caretakers (guardians) and healthcare providers. We had planned to use data from the Addis Ababa City Cancer Registry Unit (AACCRU), the only cancer registry in the country [38], for obtaining information from the patients’ caretakers (guardians). Our strategy was to identify patients who had abandoned their treatment by telephonic screening and then conduct follow-up home visit interviews. Accordingly, the AACCRU tried to contact all the patients diagnosed with childhood cancer and registered (between 1 July 2019 and 30 June 2021) to understand their treatment status and ask them if they were willing to participate in the study. However, 70 out of the 186 eligible patients’ caretakers (37.6%) were not accessible through the phone for various reasons: non-working lines, wrong numbers, or change in phone numbers. Among those who were accessible through calls, 58 stated that their child was currently receiving treatment, 47 reported that their child had died while on treatment, 2 reported completion of treatment (with the child having survived), and 9 stated that the patient had abandoned care. Seven out of the nine agreed to participate in the study. However, the number of inaccessible guardians was too high to obtain representative data. As a result, we decided to conduct the study by using the healthcare providers’ perspective only.

### Data collection and analysis

We used a validated, semi-structured questionnaire developed by the International Society of Pediatric Oncology Abandonment Technical Working Group, and previously used in a global abandonment estimate survey [14]. The questionnaire mainly covered incidence of childhood cancer treatment abandonment; influencing risk factors; availability of essential childhood cancer control interventions and strategies to reduce childhood cancer abandonment (Table S1). We administered the questionnaire in English, by using tablets and a central server. The field supervisors and the principal investigator had real-time access to de-identified data and provided feedback to data collectors whenever they identified gaps. Trained data collectors conducted in-depth face-to-face interviews from September 5 to 22, 2021. We conducted descriptive analysis using Stata/SE 17.0 version.

## Results

### Background characteristics of the study participants

Table 1 presents the background characteristics of the respondents. Thirty-eight healthcare providers from three out of four pediatric oncology centers in the country participated in our study. From the three pediatric oncology centers, all physicians (*n* = 7) and social workers (*n* = 8), and 23 out of 42 nurses were eligible for the study, and all of them agreed to participate (response rate, 100%). Most respondents (44%) were from TASH pediatric oncology center, and nurses comprised the highest proportion of participants (60%). Five out of the seven pediatric hematologist-oncologists in the country participated in the study. Further, 66% of the respondents were male. The average overall work experience in childhood cancer-related services was 3.2 years: TASH had the highest average work experience (4 years), and it was followed by JUH (3.2 years) and GUH (2.5 years). At an individual level of observation, work experience ranged from 2 to 13 years. The average number of new cases per year, as estimated by the respondents since there were no robust cancer registries, was the highest in TASH (754 patients), and JUH and GUH had an estimated 119–122 new cases per year. Government financing is the major funding source at all centers, and this is followed by out-of-pocket payment by guardians. In addition, there are a few implementing partners and civil society organizations that provide social support for people in need. These organizations work closely with TASH and, to some extent, with JUH.

**Table 1:** General background characteristics of the respondents

Variables		Name of the hospital			
		Tikur Anbessa Specialized Hospital, N (%)	Gondar University Hospital, N (%)	Jimma University Hospital, N (%)	Total, N (%)
Total healthcare professionals	Physician	3 (42.8)	3 (42.8)	1 (14.6)	7 (100.0)
	Nurse	23 (54.7)	10 (23.8)	9 (21.4)	42 (100.0)
	Social worker	3 (37.5)	3 (37.5)	2 (25.0)	8 (100.0)
	Total	29 (50.8)	16 (28.1)	12 (21)	57 (100.0)
Eligible participants	Physician	3 (42.8)	3 (42.8)	1 (14.6)	7 (100.0)
	Nurse	11 (47.8)	6 (26.1)	6 (26.1)	23 (100.0)
	Social worker	3 (37.5)	3 (37.5)	2 (25.0)	8 (100.0)
	Total number of participants	17 (44.7)	12 (31.58)	9 (23.7)	38 (100.0)
Physician	Pediatric hematologist-oncologist	3	1	1	5
	Pediatrician	0	1	0	1
	Resident	0	1	0	1
Sex: n (%) females, n (%) males		7 (41), 59 (10)	2 (17), 10 (83)	4 (44), 5 (56)	13 (34), 25 (66)
Work experience in childhood cancer care in years (mean, 95% confidence interval [CI])		4[2.4–5.6]	2.5[2.1–2.9]	3.2[2.1–4.3]	3.2[2.6–4.0]
Average annual number of cases (mean, 95% CI)		754[642–867]	119[104–134]	122[106–139]	426[294–559]

### Incidence of treatment abandonment and associated risk factors

The mean perceived abandonment rate was 34% (standard error (S.E) 2.5) (Table 2). The estimate was the lowest for TASH 28.3% (S.E 3.5%), while it was 40.7% (S.E 4.4%) for JUH and 40.6% (S.E 3.7%) for GUH. On an individual level, 57% of the respondents perceived the abandonment rate to be higher than 35% (Table S2).

**Table 2:** Perceived estimate of abandonment

Pediatric oncology center	Mean	Standard error (S.E)	95% CI
Tikur Anbessa Specialized Hospital	28.3%	3.5%	21.2–35.5%
Gondar University Hospital	40.6%	3.7%	33–48%
Jimma University Hospital	40.7%	4.4%	31.4–49.8%
Overall	34.7%	2.5%	29.7–39.7%

**Fig. 1:**
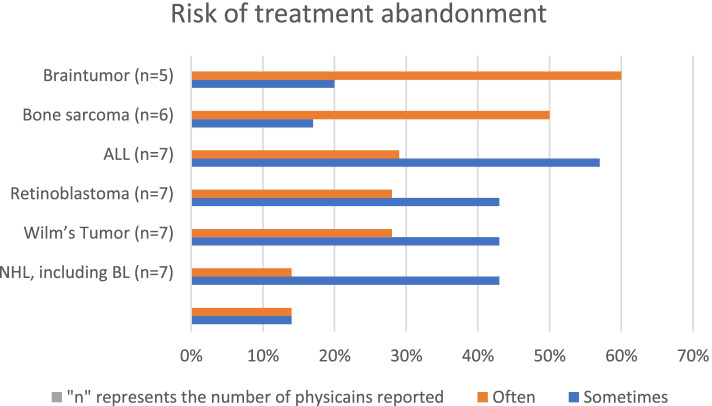
Risk of treatment abandonment by childhood cancer type

We asked physicians to report the risk of abandonment related to the commonly reported childhood cancers in Ethiopia by using the following categories: never, rarely, sometimes, often, always, don’t know. The “don’t know” responses were not included in the analysis, and the “often” and “always” responses were aggregated to indicate a high risk of abandonment. The perceived risk of abandonment was relatively higher for brain tumor and bone sarcoma (the most frequent response was “often”), and lower for non-Hodgkin’s lymphoma and Hodgkin’s lymphoma (Fig. 1).

**Fig. 2:**
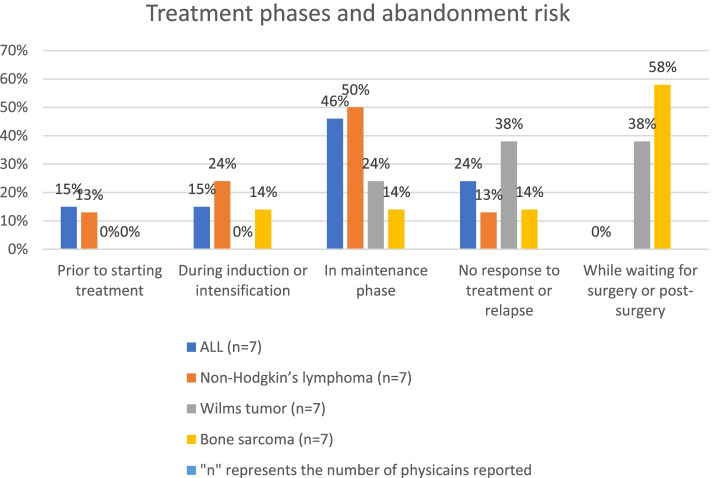
Abandonment risk associated with childhood cancer treatment phases and outcomes

We asked physicians to indicate the treatment phase or outcome (pre-treatment, induction/intensification, maintenance, no response to treatment/relapse, other) that carried the highest risk of abandonment for selected common childhood cancers (representing leukemia, lymphoma and solid tumor) in Ethiopia and allowed them to choose more than one option, if needed (Fig. 2). According to the physicians, patients with acute lymphoblastic leukemia (ALL) are highly likely to abandon care in the maintenance phase of the treatment cycle (46%). Children with Wilms tumor (38%) or bone sarcoma (58%) are highly likely to abandon treatment while waiting for surgery or in the post-surgery period. Additionally, failure to respond to treatment or relapse was estimated as a high-risk factor for treatment abandonment in cases of Wilms tumor and ALL. Further, close to 15% of ALL and non-Hodgkin’s lymphoma patients abandoned care before the start of treatment (Fig. 2). Finally, 72% of the respondents reported that there is no routine practice for tracing defaulters as there is no contact tracing mechanism (Table S3).

**Fig. 3:**
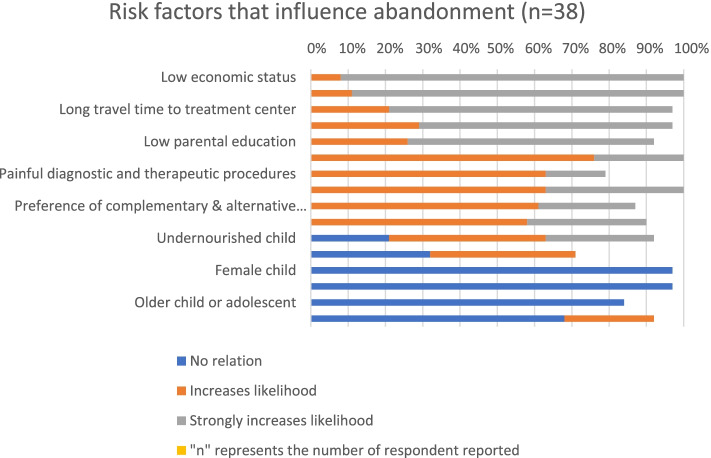
Risk factors associated with treatment abandonment

We asked participants to indicate the level of influence of certain pre-identified risk factors on treatment abandonment at their center (Table S4). These risk factors were previously identified by the International Society of Pediatric Oncology Abandonment Technical Working Group [14]. We asked them to grade the factors based on their likelihood of leading to abandonment as follows: strongly decrease likelihood, decrease likelihood, no relation, increase likelihood, strongly increase likelihood. At the analysis stage, we developed five categories (major role, important role, moderate role, minor role, and no relation) based on a combination of reported responses on the likelihood of abandonment (Supplementary text S 1 summarizes how each category was constructed). The healthcare providers reported that low economic status, high cost of care (related to diagnostics, chemotherapy, radiotherapy, surgery, supportive care, food, and lodging), long travel time to the treatment center, belief in the incurability of cancer, and low level of parental education played a major role in treatment abandonment (Fig. 3). Undernourishment, adverse effects and toxicity of treatment, painful diagnostic and therapeutic procedures, insufficient communication by healthcare professionals, preference for complementary and alternative medicine, and strongly held faith or religious beliefs were found to play an important role in influencing treatment abandonment, while HIV diagnosis and younger age of the child played a moderate and minor role, respectively. The sex of the child and older age of the child or adolescence were perceived as having no relationship with abandonment.

The likelihood of healthcare providers accepting a guardian’s decision to abandon care was affected by the clinical prognosis of patients (see Table S3). Two out of seven (29%) physicians reported they would accept the decision of guardians to abandon care without further discussion, if the guardian of a child with a bad prognosis refused to start treatment. Four out of seven physicians (57%) reported that they would accept the decision under these circumstances if the guardian refused to continue treatment. On the other hand, in the case of children who had a good prognosis, all seven physicians reported they would either counsel guardians to convince them (to start or continue treatment) or connect them with social workers who could assist them (see Table S3).

### Availability of essential interventions for the treatment of childhood cancer

We asked the physicians and nurses to evaluate the availability of essential childhood cancer treatment-related interventions at their centers (Table S5). The perceived availability of social support, free/subsidized food and blood product scored greater than 95% while availability of effective procedural sedation and analgesia, free or subsidized chemotherapy and surgery scored 75–80%; free/subsidized lodging 67%, and financial support for travel and availability of locally adopted treatment protocol 53%. Lodging and financial support for travel were not available at GUH. It is important to note that these perceived availability estimates only indicate the system-level willingness to address the barriers of childhood cancer treatment. However, it does not reflect the actual uninterrupted availability of these interventions. For example, frequent stockout of chemotherapy supplies was mentioned as one of the contributing factors for treatment abandonment in the open-ended questions, even though the system-level availability of subsidized chemotherapy was perceived as being high (77%).

### Strategies to reduce the abandonment of childhood cancer treatment

We asked the healthcare providers to what extent the availability of globally recommended essential pediatric oncology treatment-related interventions might reduce abandonment in their setting, and we also asked an open-ended question so that they could suggest additional strategies or interventions to reduce abandonment. According to their responses, the provision of free/subsidized surgery, blood products, chemotherapy and other supportive drugs and supplies, lodging, food, social support, financial support for travel, establishing satellite centers, and detailed and repeated counseling had a high likelihood of reducing the treatment abandonment rate. Additionally, effective procedural sedation and analgesia and locally adopted treatment protocols were moderately likely to reduce abandonment (Table 3).

**Table 3:** Interventions to reduce the incidence of treatment abandonment

Interventions	High likelihood, n (%)	Moderate likelihood, n (%)	Minimal likelihood, n (%)	Total, N (%)
Free/subsidized surgery	38 (100)			38 (100)
Free/subsidized blood products	38 (100)			38 (100)
Free/subsidized chemotherapy	38 (100)			38 (100)
Free/subsidized lodging	38 (100)			38 (100)
Social support	37 (97)	1 (3)		38 (100)
Financial support for travel	37 (97)	1 (3)		38 (100)
Free/subsidized food	36 (94)	1 (3)	1 (3)	38 (100)
Free/subsidized supportive care drugs, e.g., antibiotics	36 (94)	2 (6)		38 (100)
Development of a satellite center	35 (92)	3 (8)		38 (100)
Detailed and repeated counseling	32 (84)	6 (16)		38 (100)
Effective procedural sedation and analgesia	25 (66)	9 (23)	1 (3)	38 (100)
Locally adopted treatment protocols	15 (39)	12 (32)	5 (13)	38 (100)

Our of the 38 participants, 30 responded to the open-ended questions and reported that the following interventions would play an important role in reducing treatment abandonment: increasing government focus on the program, building human resource capacity (in terms of number of personnel and skill diversification such as hematologist-oncologists, oncology nurses, pharmacists, pathologists, and nutritionists) through short-term and long-term training, improving public awareness, improving diagnostic capacity and stockout of chemotherapy supplies, establishing a child-friendly environment (with engaging activities and motivation mechanisms), providing special foods to help children go through therapy, increasing senior physicians’ (pediatric hematologist-oncologist) engagement with patients (most children were treated and followed up by a pediatric resident who has lesser training than the senior physician); providing health insurance coverage, establishing a multidisciplinary team, and establishing a contact tracing mechanism (Table 4).

**Table 4:** Interventions proposed to decrease treatment abandonment (findings from the qualitative question)

Additional factors that could improve treatment abandonment	Frequency of reporting, n (%)
Improving government focus on the program^a^	13 (43%)
Short-term training and orientation for health professionals working at different levels, and increasing the number of pediatric hematologist-oncologists, oncology nurses, pharmacists, phycologists, pathologists, and nutritionists	13 (43%)
Creating public awareness about the curability of cancer and its early signs	11 (37%)
Improving diagnostic capacity to avoid delays in diagnosis, misdiagnosis, and mistreatment	9 (30%)
Providing special foods (that are different from that given to other patients) that could help patients go through therapy better	8 (27%)
Establishing a child-friendly environment	7 (23%)
Reducing stockout of chemotherapy supplies	6 (20%)
Improving senior physicians’ (pediatric hematologist-oncologist) follow up and contact time with patients (most children are followed by a resident or pediatrician)	6 (20%)
Improving the linkage of childhood cancer services with health insurance	4 (13%)
Establishing a multidisciplinary team to improve service quality^b^	3 (10%)
Establishing a contact tracing mechanism	3 (10%)

^a^Allocating adequate budget, human resource training, establishing diagnostic centers, improving the availability of drugs and supplies, providing equipment such chemotherapy machines, and periodic monitoring

^b^ Multidisciplinary team: includes (but is not limited to) pediatric oncologists, nurses, pharmacists, pathologists, surgeons, radiologists, respiratory therapists, anesthesiologists, social workers, and data clerks

## Discussion

The estimated childhood cancer treatment abandonment rate in Ethiopia is 34% (S.E 2.5%). The estimate is lower for TASH (28.3%, S.E 3.5%) than for JUH (40.7%, S.E 4.4%) and GUH (40.6%, S.E 3.7%). This difference might be related to the relatively better availability of drugs, diagnostic services, beds, and trained personnel at TASH, as well as the social support (food and lodging, transport, and investigation and drug expenses) provided by civil society organizations. Although our estimate is based on health care providers’ opinions and is not an actual estimate from the registry, our finding is similar to previous registry-based estimates reported for Uganda (32%), Sudan (35%), Ghana (42%) and Zambia (45.7%) [20, 22, 25–28]. However, it is higher than the estimate in Malawi (19%) [29] and lower than the estimate in Kenya (50%–54%) [21, 23].

The highest risk for treatment abandonment varies by type of cancer (high for bone sarcoma and brain tumor) and the phase and outcome of treatment. In the case of ALL, the highest risk is in the maintenance phase, or if the patient didn’t respond to treatment or relapse. The high-risk time for Wilms tumor and bone sarcoma is while waiting for surgery or post-surgery. Similar findings have been reported in the global abandonment survey using healthcare providers’ perspective [14] and a study in Sudan showed that close to 35% patients with Wilms tumor abandoned treatment prior to surgery [27]. The high risk of abandonment in the maintenance phase could be mainly related to misunderstanding of the early-stage improvement as a cured child (false sense of security) but also be related to prolonged treatment, computing household priorities, cost of care and financial hardship. The high risk of abandonment when there was no response to treatment or during relapse could be explained by loss of hope due to the poor prognosis communication, and associated preference to complementary and alternative medicines. It can also be related to cross cutting challenge like cost of care and financial hardship, computing social priorities. The high risk of abandonment while waiting for surgery or in the post-surgery period could be related to a long waiting time for surgery or radiotherapy, poor communication between departments, fear of the surgical outcome (e.g., fear of post-surgery functional impairment such as loss of vision and amputation) [29], and lack of a patient tracing mechanism. Overall, our findings indicate the need to understand and address cancer-specific abandonment-related factors, apart from general system-level risk factors indicated below.

**Fig. 4:**
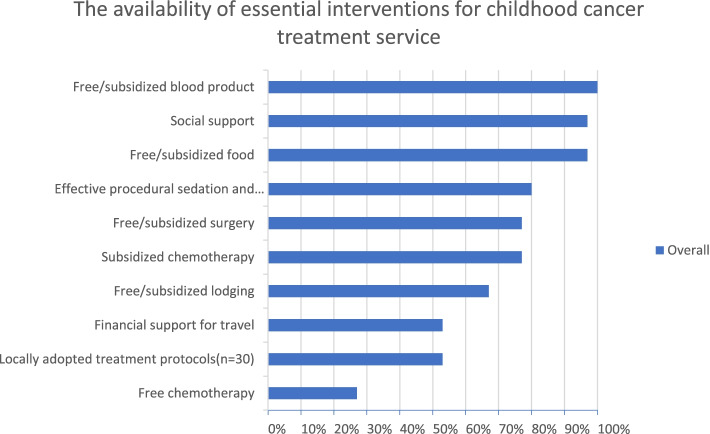
Availability of essential childhood cancer treatment interventions at oncology centers

The healthcare providers in the present study reported that the following health system (supply side) and community (demand side) barriers influence abandonment. The perceived supply-side barriers were as follows: high cost of care; limited physical and effective access to pediatric oncology services (reflected in long travel time, long waiting time for surgery and radiotherapy, interrupted supply of chemotherapy, and poor availability of diagnostic services); suboptimal human resource capacity, and suboptimal care by existing hematologist-oncologists; poor pain and toxicity management; poor prognosis at the time of diagnosis or treatment; poor rapport between the patient/guardian and clinician; lack of food that is tailored to the needs of children on cancer therapy, and lack of food and lodging support for guardians; lack of a child-friendly environment; low insurance coverage; and lack of contact tracing mechanisms. In particular, prediction of poor prognosis at the time of diagnosis or during treatment seems to highly influence the efforts of physicians in terms of providing counseling, convincing patients and their guardians, or connecting them to social workers for support. Such effort on the part of physicians might influence guardians to change their mind and continue with treatment, and this could have high impact in Ethiopia, given that most patients are diagnosed at a very late stage [27, 39]. The lack of contact tracing mechanisms is another key factor fueling treatment abandonment, as there is no way to counsel and return patients to care once they abandon care. The findings show there is a policy level attention and enabling situation to avail pediatric cancer services at affordable price (as most interventions are planned to be delivered as either free or subsidized cost) (Fig. 4 and Table 3) but the challenge is the low effective access related to suboptimal resource allocation that doesn’t match the needs. The perceived demand-side barriers were as follows: low economic status, poor public awareness (about the curability of childhood cancer and its early signs), low level of parental education, preference for complementary and alternative medicine, and strong faith or religious beliefs.

The findings of this study are similar to reported risk factors influencing abandonment in other LMICs [14, 15, 21, 23–25, 30, 40], but the degree of influence differs. That is, even though our findings are consistent with the global survey findings for sub-Saharan Africa (which includes Ethiopia, Kenya, Tanzania, Rwanda, Mozambique, Mali, Malawi, South Africa, and Nigeria, for example) that were obtained using similar methods, the perceived risk of low economic status, cost of care, long travel time to treatment centers, belief in the incurability of cancer, and low level of parental education contributing to abandonment in Ethiopia is much higher according to our estimate. This could reflect the context-specific nature of the influencing risk factors and the need for context-specific prioritization of mitigation strategies.

Prioritizing the most influential risk factors is key, given that there are too many risk factors that are complex and require time to address. Importantly, mitigating some of these risk factors might even be beyond the scope of the health sector. Therefore, more emphasis should be placed on risk factors that play a major role, and mitigation strategies that are high-impact, affordable, and feasible for implementation should be prioritized. It is important to have a system in place that can progressively but substantially reduce the treatment abandonment rate. Thus, the development of high-impact, affordable, and feasible strategies to reduce the effect of the major risk factors (that is, low economic status and poor public awareness in terms of demand-side barriers, and high cost of care, long travel time, long waiting time, and interrupted availability of services in terms of supply-side barriers) is critical to achieving the ambitious goal set in the NCACCP (to reduce the rate of abandonment by 60% by 2023) [35]. To achieve this conducting a comprehensive situation analysis and system readiness assessment; identifying high impact and affordable interventions and developing short, medium, and long-term strategies to mitigate abandonment is critical.

There is growing evidence that public financing of childhood cancer control programs is cost effective, affordable, feasible, and sustainable [13], and the recently launched WHO Global Initiative for Childhood Cancer advises countries to prioritize these programs in their national health policies. Apart from the affordability, effectiveness, and feasibility of childhood cancer control programs, they are important from the viewpoint of equity, human rights, and social justice [13]. In the Ethiopian context, various options can be explored to address the major influential risk factors. Ethiopia has a long standing experience in exempting payment for high-priority health services [41]. Accordingly, the inclusion of childhood cancer control interventions in the exemption list and the allocation of additional funds towards it can be considered. This could have a strong impact on resolving the economic barriers that lead to abandonment such as high cost of care, low-income status, and limited access to care. Addressing the cost related barrier will be important since it is the highly reported influencing risk factor, and reducing cost of care (as an exemption or subsidy) is the most recommended strategy—by the health professionals in our study, to improve abandonment. In the long run, the health service exemption needs to be supplemented with sustainable forms of alternative financing, such as the Community-Based Health Insurance (CBHI) program. To achieve this, the population coverage of the CBHI program (which currently covers 56% of the population) needs to be expanded [42], and more importantly, a mechanism needs to be devised to cover the cost of care at pediatric oncology treatment centers for those who are already enrolled. Currently, even though tertiary-level care, including oncology treatment, is covered by the CBHI benefit package, in reality, CBHI is highly restricted to district-level primary healthcare services. That is, it does not cover tertiary care, which requires patients to travel outside of their district or regions, as is true for almost all cases of children with cancer. Most CBHI schemes have contracts with health facilities in their district only, and there is no robust system, that allows the coverage of services are not available in the patients’ district or regions [43] since there is no higher level fund pooling arrangement or alternative temporary mechanism to ensure continuity of service for CBHI beneficiaries. Mobilization of additional resources through development assistance (for example, advocacy for global partners to support childhood cancer control programs), twinning of local oncology centers with international centers (for drug and supply support, long- and short-term training of human resources, and standardization of care), and encouraging international and local civil society organizations to provide additional social support (such as support for food, lodging, and transport) are also important measures to tackle the issue of treatment abandonment. Given the promising high survival rate (> 80% in HICs) of childhood cancer to timely and quality therapy [13, 44] and the time-sensitive nature of treatment (better survival rate in the early cancer stage) [13], a potentially beneficial strategy might be to prioritize childhood cancer in diagnostic, radiotherapy, and surgical schedules, in contrast to the first-come-first-serve approach that is currently being practiced. To realize this, there is a need to develop clinical care prioritization guidelines through a participatory and transparent process. Such a measure could reduce the long waiting time to diagnosis and treatment and abandonment rate and, thereby, improve the survival rate of childhood cancer patients. To address the frequent stockout of drugs and supplies, childhood cancer drugs and supplies can be included in the national essential drug lists and follow-up lists of key procurement performance indicators; additionally, the long-term procurement framework and the financing source and mechanism need to be clarified. Another strategy is networking between pediatric oncology centers and other hospitals (based on geographical distribution) that can function as satellite sites, as this could address the barrier of limited physical access to treatment services.

Demand-side barriers, such as poor public awareness, preference for complementary and alternative medicine, and strong faith or religious beliefs, can be addressed through continuous awareness creation and the engagement and empowerment of community and other stakeholders. With regard to the perceived preference for complementary and alternative medicine and incurability of childhood cancer, some beneficial strategies are preparing a tool for community conversation, building the capacity of community health workers (health extension workers in the case of Ethiopia), and mapping and targeting key community influencers associated with abandonment (such as traditional healers, religious leaders, and village or clan leaders). In addition, continuous awareness creation using mass media, providing financial support for travel, and other community-driven social support initiatives could help reduce the treatment abandonment rate. These measures could be feasible and affordable given Ethiopia’s extensive experience in health promotion and disease prevention at the primary healthcare level and the longstanding community structure and networking in place (for example, the Health Extension Program) [45]. Further, the growing access to media, such as radio, TV, mobile phone, and social media, in Ethiopia can facilitate the reachability of awareness creation activities [46, 47]. While placing emphasis on the highly influential risk factors, it is also important to work on improving the rapport between clinicians and patients/parents, pain management, human resource capacity (short- and long-term training), and senior physician engagement, creation of a child-friendly environment and adopting localized treatment protocols. Standardized treatment protocol can reduce treatment abandonment through minimizing the back-and-forth treatment trials and having a treatment aligned with available clinical supportive care (that can reduce associated treatment toxicity and toxicity related complications including death, cost, duration of treatment); improving patient trust and improving treatment outcome.

The key to successful implementation of the aforementioned interventions is improving government ownership (within and outside the health sector) of childhood cancer control programs; in addition, strategically planned and sustained advocacy work needs to be conducted at various levels of the government administration. This could help to translate the existing policy level attention for childhood cancer into a real commitment. Importantly, policy makers need to maximize the potential benefits of the new global movement for the support of childhood cancer control programs across the LMICs [13].

Our study has several limitations, but the major shortcoming is that we only captured the healthcare providers’ side of the story, as our efforts to include patients’ and guardians’ perspectives failed. As a result, we may have missed some key influencing factors on demand-side barriers. A mixed-methods design that includes quantitative and qualitative study about the guardian’s perspective (instead of a structured quantitative study only) would have been robust in terms of identifying risk factors, especially local context-specific risk factors. Another limitation is that the estimated abandonment rate is the perceived abandonment rate and not the actual estimate determined from the registry data; therefore, the reported abandonment rate could be an over- or underestimation of the actual rate. Further, despite the use of a validated questionnaire and rigorous quality control of the data, there is a possibility of inter-respondent variation in understanding the questionnaire. However, our findings are still relevant in terms of informing national childhood cancer control programs and augmenting global knowledge about the incidence and risk factors of abandonment of childhood cancer treatment, given that the findings are consistent with those of other studies conducted in a similar setting.

## Conclusions and recommendations

The present findings indicate that the perceived abandonment rate of childhood cancer treatment in Ethiopia is high, and is closely linked with the cancer type and phase of treatment or treatment outcome. Despite the similarity in the risk factors reported here and other studies, the level of influence varies across settings and context-specific prioritization is important.

Based on our results, we recommend that national childhood cancer programs prioritize and address the following supply- and demand-side barriers to improve the survival rate of children with cancer [1]. The proposed measures for addressing supply-side barriers are as follows: freeing/heavily subsidizing the high cost of care; ensuring uninterrupted availability of services; prioritizing children with cancer for shared hospital services (such as diagnostics, surgery, and radiotherapy) to decrease waiting time; and exploring options for establishing satellite sites. Along with addressing the high-priority risk factors, there is a need to improve the rapport between clinicians and patients/guardians and pain and toxicity management, as well as to provide special foods that can help patients tolerate the treatment process better and a child-friendly environment [2]. The following measures are proposed for addressing demand-side barriers: social support to guardians with low economic status and improving public awareness about childhood cancer. Identifying the type of cancer and treatment center-specific risk factors for abandonment, and developing specific mitigation plans are important. Establishing a contact tracing mechanism could help to identify defaulters on time and convince them to resume treatment. Increasing government and other stakeholders’ focus on and engagement with childhood cancer care is also critical to addressing the identified risk factors and in translating the existing policy level priority attention into tangible actions. Further, strengthening the cancer registry in TASH and scaling it to the other centers could be instrumental for periodically monitoring treatment outcomes, including abandonment, and facilitating timely decision making. Future similar work using a robust registry and a prospective, mixed-methods design (qualitative and quantitative study) that includes guardians’ perspective could provide a better understanding of the magnitude of the problem and the factors associated with it, especially in terms of identifying context-specific demand-side risk factors.

## Supplementary information

**Additional file 1.**

## Data Availability

The datasets used and/or analyzed during the current study are available from the corresponding author on reasonable request.
