# Supplementary material for: The magnitude and perceived reasons for childhood cancer treatment abandonment in Ethiopia: from health care providers’ perspective
Source: BMC Health Serv Res. 2022 Aug 8;22:1014. doi: 10.1186/s12913-022-08188-8 (PMC9361525; doi:10.1186/s12913-022-08188-8)
Supplement: Supplementary file 1 — Additional file 1. [file 12913_2022_8188_MOESM1_ESM.docx]

Supplementary Material

Table S1: Data collection tool for the study of childhood cancer treatment abandonment in Ethiopia

| **SECTION I: INTRODUCTION & CONSENT (IC)** | | | | |
| --- | --- | --- | --- | --- |
| **NO.** | **QUESTION** | | **RESPONSE** | **SKIP** |
| Facility name: | | | | |
| SC1 | ARE YOU A CLINICIAN, NURSE, SOCIAL WORKER INVOLVED IN THE CARE OF CHILDREN WITH CANCER? | | Yes…………………………………1  No………………………………….2 | 1 🡪 SC2  2 🡪 END |
| SC2 | HOW LONG HAVE YOU BEEN CARING FOR CHILDREN WITH CANCER IN A PROFESSIONAL CAPACITY? | | Less than 1 year……………………….1  1–2 years………………………............2  2–5 years………………………............3  More than 5 years (est): [ ] years……..4 | 1 🡪 END  2 🡪 CONS  3 🡪 CONS  4 🡪 CONS |
| **CONSENT FORM (CONS)** | | | | |
| You are invited to take part in a research study on abandonment of treatment in children with cancer. Abandonment of treatment is considered a major cause of treatment failure in children with cancer in Ethiopia, but the scientific evidence for it is limited. We (Addis Center for Ethics and Priority Setting) are, therefore, conducting this survey to study the extent of abandonment in Ethiopia, and to identify the related factors and mitigation strategies.  You are invited to join this study if you are a **doctor**, **nurse**, or **social worker** involved in the care of children with cancer. The survey will take 30 minutes to complete and will ask questions about the setting in which you work, how abandonment may affect patients, the risk factors, and strategies that may be used in your facility.  This survey is completely voluntary. Your work or relationship with any of the study’s team members will not be affected by your participation in this study. We will also respect your privacy: the data collected is confidential, and your answers will not be linked to any details that could identify you in the final study report.  By completing this survey, you consent to take part in this research study. We know of no harm that taking part in this study could cause you. You will not benefit directly from taking part in this study. | | | | |
| **Statement of Consent**  I have read the description of the research or have had it translated into a language I understand. I understand that my participation is voluntary. I know enough about the purpose, methods, risks, and benefits of the research study to judge that I want to take part in it. I understand that I may freely stop being part of this study at any time and I can ask to erase shared information. I understand that shared information will be stored in the University of Bergen server, Norway, for analysis. I have received a copy of this consent form to keep for myself. | | | | |
|  | |  | | |
| *Date* | | *Signature of Participant* | | |
| **Investigator's statement**  I, the undersigned, have explained to the volunteer in a language he/she understands the procedures to be followed in the study and the risks and benefits involved, and I have given a copy of the consent form to the participant. | | | | |
| *Address of chief investigator* | | *Email:*  *Phone number:* | | |
| *Date* | | *Signature of Participant* | | |

| **SECTION II: INTERVIEW (IN)** | | | | | | | | | | | | | | | | | | | | | | | | | | | |
| --- | --- | --- | --- | --- | --- | --- | --- | --- | --- | --- | --- | --- | --- | --- | --- | --- | --- | --- | --- | --- | --- | --- | --- | --- | --- | --- | --- |
| **NO.** | **QUESTION** | | | **RESPONSE** | | | | | | | | | | | | | | | | | | | | | | | **SKIP** |
| IN1 | WHAT IS YOUR OCCUPATION? | | | Physician..………………..……..…1  Nurse/nurse practitioner..….…...….2  Social worker………………………3  Other: ……………………………....4 | | | | | | | | | | | | | | | | | | | | | | |  |
| IN2 | IF YOU ARE A PHYSICIAN, HOW WOULD YOU BEST DESCRIBE YOURSELF? | | | Pediatric hematologist and/or oncologist…………………………1  Adult hematologist and/or oncologist…………………………2  General pediatrician……………...3  General physician………………….4  Other: _______________________ 5 | | | | | | | | | | | | | | | | | | | | | | |  |
| IN3 | WHAT IS YOUR SEX? | | | Male………………………………..1  Female……………………………..2 | | | | | | | | | | | | | | | | | | | | | | |  |
| IN4 | APPROXIMATELY HOW MANY CHILDREN NEWLY DIAGNOSED WITH CANCER (INCLUDING CHILDREN WITH LEUKEMIAS, LYMPHOMAS, SOLID TUMOURS AND BRAIN TUMOURS) ARE THERE IN YOUR CENTER OVER ONE YEAR? | | | [ ] patients per year    Collect data from the register or ask their opinion if register is not available | | | | | | | | | | | | | | | | | | | | | | |  |
| IN5 | AT YOUR CENTER, APPROXIMATELY WHAT PROPORTION OF CHILDREN DIAGNOSED WITH CANCER DIE WITHIN THE FIRST YEAR OF DIAGNOSIS | | | 20% or less………………………….1  21% to 30%........................................2  31% to 40%.........................................3  41% to 50%........................................4  51% to 65%........................................5  66% to 80%........................................6  More than 81%...................................7  Don’t know…………………..……...9 | | | | | | | | | | | | | | | | | | | | | | |  |
| IN6 | AT YOUR CENTER, APPROXIMATELY WHAT PROPORTION OF CHILDREN DIAGNOSED WITH CANCER DIE WITHIN THE FIRST TWO YEARS OF DIAGNOSIS | | | 25% or less………………………….1  26% to 40%........................................2  41% to 50%.........................................3  51% to 60%........................................4  61% to 70%........................................5  71% to 80%........................................6  More than 81%...................................7  Don’t know…………………..……...9 | | | | | | | | | | | | | | | | | | | | | | |  |
| IN7 | AT YOUR CENTER, APPROXIMATELY WHAT PROPORTION OF CHILDREN DIAGNOSED WITH CANCER DIE WITHIN THE FIRST FIVE YEARS OF DIAGNOSIS | | | 30% or less………………………….1  31% to 45%........................................2  46% to 55%.........................................3  56% to 65%........................................4  66% to 75%........................................5  76% to 85%........................................6  More than 86%...................................7  Don’t know…………………..……...9 | | | | | | | | | | | | | | | | | | | | | | |  |
| IN8 | WHAT ARE THE SOURCES OF FUNDING FOR THE CARE OF CHILDHOOD CANCER PATIENTS IN YOUR SETTING?  [1] = Major source  [2] = Minor source  ***Circle all that apply*** | | | Government (tax or insurance)…………………..[1]…[2]  Private insurance…………...[1]…[2]  Out-of-pocket payment by patient/family………………[1]…[2]  National non-profit organization………………..[1]…[2]  International non-profit organization………………..[1]…[2]  Do not know……………………….9 | | | | | | | | | | | | | | | | | | | | | | |  |
| IN9a | AT YOUR CENTER, APPROXIMATELY WHAT PROPORTION OF CHILDREN NEWLY DIAGNOSED WITH CANCER ABANDON TREATMENT (INCLUDING THOSE WHO ABANDON CARE EVEN BEFORE TREATMENT IS STARTED)? | | | 15% or less…………………………1  16% to 25%......................................2  26% to 35%......................................3  36% to 45%......................................4  46% to 55%......................................5  56% to 65%......................................6  66% to 75%......................................7  More than 75%.................................8  Don’t know………………………...9 | | | | | | | | | | | | | | | | | | | | | | |  |
| IN9b | From the categories you selected in IN9a, what is your average estimate for abandonment | | | __________ % | | | | | | | | | | | | | | | | | | | | | | |  |
| IN10 | WHERE DOES THIS ESTIMATE COME FROM? WE VALUE ALL RESPONSES EQUALLY, WHETHER THEY COME FROM A DATABASE OR FROM YOUR PERSONAL EXPERIENCE. | | | Personal opinion, I feel confident….1  Personal opinion, but not confident…2  Estimate comes from a database…….3 | | | | | | | | | | | | | | | | | | | | | | |  |
| IN11 | FOR EACH OF THE FOLLOWING CHILDHOOD CANCERS, HOW LIKELY IS TREATMENT ABANDONMENT IN YOUR FACILITY? (for physicians only) | | | | | | | | | | | | | | | | | | | | | | | | | | |
|  | Type of cancer | | | Never/Almost Never | | | | | Rarely | | | | | Some-times | | Often | | | | | Always/Almost always | | | | | Don’t know | |
|  | Acute lymphoblastic leukemia (ALL) | | |  | | | | |  | | | | |  | |  | | | | |  | | | | |  | |
|  | Acute myeloid leukemia | | |  | | | | |  | | | | |  | |  | | | | |  | | | | |  | |
|  | Hodgkin’s lymphoma | | |  | | | | |  | | | | |  | |  | | | | |  | | | | |  | |
|  | Non-Hodgkin’s lymphoma (including Burkitt’s lymphoma) | | |  | | | | |  | | | | |  | |  | | | | |  | | | | |  | |
|  | Brain tumors | | |  | | | | |  | | | | |  | |  | | | | |  | | | | |  | |
|  | Wilms Tumor | | |  | | | | |  | | | | |  | |  | | | | |  | | | | |  | |
|  | Retinoblastoma | | |  | | | | |  | | | | |  | |  | | | | |  | | | | |  | |
|  | Soft tissue sarcoma | | |  | | | | |  | | | | |  | |  | | | | |  | | | | |  | |
|  | Bone sarcoma | | |  | | | | |  | | | | |  | |  | | | | |  | | | | |  | |
| Type of cINIaIN12 | HOW LIKELY IS CHILDHOOD CANCER TREATMENT ABANDONMENT IN YOUR FACILITY? (Non-Physicians) | | | Never/Almost Never | | | | Rarely | | | Some-times | | | | | | Often | | | | | Always/Almost always | | | | | Don’t know |
|  |  |  |  |  | | | |  | | | |  | | | | | |  | | | | |  | | | |  |
| IN13 | IN YOUR CENTER, AT WHAT STAGE OF TREATMENT ARE CHILDREN WITH **ACUTE LYMPHOBLASTIC LEUKEMIA** HIGHLY LIKELY TO ABANDON TREATMENT?  ***(SELECT UP TO 3 OPTIONS)*** | | | Prior to starting treatment……….…1  During induction or intensification..2  In maintenance……………….....…3  If not responding to treatment or relapsing after treatment…………………….……..4  Other: _______________________ 5  Don’t know….……………………..9 | | | | | | | | | | | | | | | | | | | | | | |  |
| IN14 | IN YOUR CENTER, AT WHAT STAGE OF TREATMENT ARE CHILDREN WITH **NON-HODGKIN’S LYMPHOMA** HIGHLY LIKELY TO ABANDON TREATMENT?  ***(SELECT UP TO 3 OPTIONS)*** | | | Prior to starting treatment……….…1  During induction or intensification..2  In maintenance……………….....…3  If not responding to treatment or relapsing after treatment…………………….……..4  Other: _______________________ 5  Don’t know….……………………..9 | | | | | | | | | | | | | | | | | | | | | | |  |
| IN15 | IN YOUR CENTER, AT WHAT STAGE OF TREATMENT ARE CHILDREN WITH **WILMS TUMOR** HIGHLY LIKELY TO ABANDON TREATMENT?  ***(SELECT UP TO 3 OPTIONS)*** | | | Prior to starting treatment……….…1  During induction or intensification..2  In maintenance……………….....…3  If not responding to treatment or relapsing after treatment…………………….……..4  Other: _______________________ 5  Don’t know….……………………..9 | | | | | | | | | | | | | | | | | | | | | | |  |
| IN16 | IN YOUR CENTER, AT WHAT STAGE OF TREATMENT ARE CHILDREN WITH **BONE SARCOMAS** HIGHLY LIKELY TO ABANDON TREATMENT?  ***(SELECT UP TO 3 OPTIONS)*** | | | Prior to starting treatment……….…1  During induction or intensification..2  In maintenance……………….....…3  If not responding to treatment or relapsing after treatment…………………….……..4  Other: _______________________ 5  Don’t know….……………………..9 | | | | | | | | | | | | | | | | | | | | | | |  |
| IN17 | HOW ARE THE FOLLOWING FACTORS RELATED TO THE LIKELIHOOD OF ABANDONMENT IN YOUR FACILTY ? | | | | | | | | | | | | | | | | | | | | | | | | | | |
|  | Factor | | Strongly decreased likelihood | | | Decreased likelihood | | | | No relation | | | | | Increased likelihood | | | | | Strongly increased likelihood | | | | | Don’t know | | |
|  | Younger age of the child | |  | | |  | | | |  | | | | |  | | | | |  | | | | |  | | |
|  | Older age of the child or adolescence | |  | | |  | | | |  | | | | |  | | | | |  | | | | |  | | |
|  | Female sex | |  | | |  | | | |  | | | | |  | | | | |  | | | | |  | | |
|  | Male sex | |  | | |  | | | |  | | | | |  | | | | |  | | | | |  | | |
|  | Undernourishment | |  | | |  | | | |  | | | | |  | | | | |  | | | | |  | | |
|  | HIV diagnosis of the child | |  | | |  | | | |  | | | | |  | | | | |  | | | | |  | | |
|  | Low level of parental education | |  | | |  | | | |  | | | | |  | | | | |  | | | | |  | | |
|  | Low socioeconomic status | |  | | |  | | | |  | | | | |  | | | | |  | | | | |  | | |
|  | Long travel time to center | |  | | |  | | | |  | | | | |  | | | | |  | | | | |  | | |
|  | Adverse effects and toxicity of treatment | |  | | |  | | | |  | | | | |  | | | | |  | | | | |  | | |
|  | Painful diagnostic and therapeutic procedures | |  | | |  | | | |  | | | | |  | | | | |  | | | | |  | | |
|  | Insufficient communication by healthcare professionals | |  | | |  | | | |  | | | | |  | | | | |  | | | | |  | | |
|  | Preference for complementary and alternative medicine | |  | | |  | | | |  | | | | |  | | | | |  | | | | |  | | |
|  | Strong faith or religious beliefs | |  | | |  | | | |  | | | | |  | | | | |  | | | | |  | | |
|  | Belief in the “incurability” of cancer | |  | | |  | | | |  | | | | |  | | | | |  | | | | |  | | |
| IN18 | ARE THERE ANY OTHER FACTORS RELATED TO ABANDONMENT IN YOUR SETTING? | | | | | | | | | | | | | | | | | | | | | | | | | | |
|  | Factor 1: |  | | | | | | | | | | | | | | | | | | | | | | | | | |
|  | Factor 2: |  | | | | | | | | | | | | | | | | | | | | | | | | | |
|  | Factor 3: |  | | | | | | | | | | | | | | | | | | | | | | | | | |
| IN19 | FOLLOWING DIAGNOSIS, IF A CHILD WITH A CANCER HAVING A GOOD PROGNOSIS (E.G., STANDARD-RISK ACUTE LYMPHOBLASTIC LEUKAEMIA OR HODGKIN’S LYMPHOMA) IS OFFERED TREATMENT AND FAMILY/CARERS REFUSE TO INITIATE IT, WHICH OF THE FOLLOWING WOULD OCCUR IN YOUR SETTING? | | | Decision accepted without discussion…………………………1  Family would be counselled to investigate reason/convince decision maker to change decision……………………………2  Connect with social worker………..3  Other: ______________________ 4  Don’t know…………………………9 | | | | | | | | | | | | | | | | | | | | | | |  |
| IN20 | IN CASE OF REFUSAL TO INITIATE TREATMENT FOR A CHILD WITH CANCER HAVING A POOR PROGNOSIS (E.G., METASTATIC BONE OR SOFT TISSUE SARCOMAS, AND HIGH-RISK NEUROBLASTOMA) WHICH OF THE FOLLOWING WOULD OCCUR IN YOUR SETTING? | | | Decision accepted without discussion…………………………1  Family would be counselled to investigate reason/convince decision maker to change decision…………………………….2  Connect with social worker………..3  Other: ______________________ 4  Don’t know………………………...9 | | | | | | | | | | | | | | | | | | | | | | |  |
| IN21 | IF THE FAMILY/CARER OF THE CHILD WITH A CANCER HAVING A GOOD PROGNOSIS (E.G., STANDARD-RISK ACUTE LYMPHOBLASTIC LEUKAEMIA AND HODGKIN’S LYMPHOMA) AND UNDERGOING TREATMENT REFUSES TO CONTINUE TREATMENT, WHICH OF THE FOLLOWING WOULD OCCUR IN YOUR SETTING? | | | Decision accepted without discussion…………………………1  Family would be counselled to investigate reason/convince decision maker to change decision…………………………….2  Connect with social worker………..3  Other: ______________________ 4  Don’t know………………………...9 | | | | | | | | | | | | | | | | | | | | | | |  |
| IN22 | IN CASE OF REFUSAL TO CONTINUE TREATMENT FOR A CHILD WITH CANCER HAVING A POOR PROGNOSIS (E.G., METASTATIC BONE OR SOFT TISSUE SARCOMAS, AND HIGH-RISK NEUROBLASTOMA) WHICH OF THE FOLLOWING WOULD OCCUR IN YOUR SETTING? | | | Decision accepted without discussion…………………………1  Family would be counselled to investigate reason/convince decision maker to change decision…………………………….2  Connect with social worker………..3  Other:……………………………. 4  Don’t know………………………...9 | | | | | | | | | | | | | | | | | | | | | | |  |
| IN23 | DURING ONGOING TREATMENT, IF A CHILD MISSES A SCHEDULED APPOINTMENT FOR CHEMOTHERAPY, RADIOTHERAPY, OR SURGERY, WHICH OF THE FOLLOWING WOULD OCCUR IN YOUR SETTING IN THE **FIRST INSTANCE**? | | | It is not routine practice to contact the child’s family/caretaker………………………………....1  Child’s family/caretaker contacted only if they still don’t turn up for the next few days…………………………………….….…2  Child’s family/caretaker contacted on the same/next day………………………………..3  Other: ……………………………………… 4  Don’t know……………..…………………...9 | | | | | | | | | | | | | | | | | | | | | | |  |
| IN 24 | EVALUATE THE AVAILABILITY OF THE FOLLOWING INTERVENTIIONS/STRATEGIES  1 = Available  2 = Not available  9 = Don’t know | | | Locally adopted treatment protocols ….………[___]  Effective procedural sedation and analgesia…..[___]  Free chemotherapy………………..…………...[___]  Subsidized chemotherapy ……………...……...[___]  Free/subsidized surgery…………...........……...[___]  Free/subsidized blood component therapy..…...[___]  Financial support for travel…………...………..[___]  Free/subsidized food……………….……...…...[___]  Free/subsidized lodging…………...…………...[___]  Social support……………………………….…[___] | | | | | | | | | | | | | | | | | | | | | | |  |
| IN25 | HOW LIKELY ARE THE FOLLOWING STRATEGIES TO REDUCE ABANDONMENT IN YOUR CENTER? | | | | | | | | | | | | | | | | | | | | | | | | | | |
|  | Strategy | | | | | | Very likely | | | | | | Moderately likely | | | | | | Minimally likely | | | | | Don’t know | | | |
|  | Locally adopted treatment protocols | | | | | |  | | | | | |  | | | | | |  | | | | |  | | | |
|  | Effective procedural sedation and analgesia | | | | | |  | | | | | |  | | | | | |  | | | | |  | | | |
|  | Free/subsidized chemotherapy | | | | | |  | | | | | |  | | | | | |  | | | | |  | | | |
|  | Free/subsidized supportive care drugs, e.g., antibiotics | | | | | |  | | | | | |  | | | | | |  | | | | |  | | | |
|  | Free/subsidized blood component therapy | | | | | |  | | | | | |  | | | | | |  | | | | |  | | | |
|  | Free/subsidized surgery | | | | | |  | | | | | |  | | | | | |  | | | | |  | | | |
|  | Development of a satellite center | | | | | |  | | | | | |  | | | | | |  | | | | |  | | | |
|  | Money for travel | | | | | |  | | | | | |  | | | | | |  | | | | |  | | | |
|  | Subsidy for food | | | | | |  | | | | | |  | | | | | |  | | | | |  | | | |
|  | Support for lodging, e.g., guest house | | | | | |  | | | | | |  | | | | | |  | | | | |  | | | |
|  | Patient/parent support group | | | | | |  | | | | | |  | | | | | |  | | | | |  | | | |
|  | Patient/parent information sheets | | | | | |  | | | | | |  | | | | | |  | | | | |  | | | |
|  | Detailed and repeated counselling | | | | | |  | | | | | |  | | | | | |  | | | | |  | | | |
| IN26 | ARE THERE **ANY OTHER** STRATEGIES WHICH COULD BE IMPLEMENTED IN YOUR SETTING IN THE **FUTURE** TO REDUCE ABANDONMENT? | | | | | | | | | | | | | | | | | | | | | | | | | | |
|  | Strategy 1 |  | | | | | | | | | | | | | | | | | | | | | | | | | |
|  | Strategy 2 |  | | | | | | | | | | | | | | | | | | | | | | | | | |
|  | Strategy 3 |  | | | | | | | | | | | | | | | | | | | | | | | | | |
| IN27 | WOULD YOU LIKE TO KNOW THE RESULTS OF THE SURVEY? | | | | Yes (enter email below):  [________________________________]  No | | | | | | | | | | | | | | | | | | |  | | | |
| IN28 | DO YOU HAVE ANY ADDITIONAL COMMENTS ABOUT ABANDONMENT OF TREATMENT IN CHILDREN WITH CANCER, OR ABOUT THIS SURVEY? | | | |  | | | | | | | | | | | | | | | | | | |  | | | |

Table S2. Perceived treatment abandonment rate by pediatric oncology treatment centers

| Abandonment rate | Name of hospital | | | |
| --- | --- | --- | --- | --- |
|  | Tikur Anbessa Specialized Hospital , n (%) | Gondar University Hospital, n (%) | Jimma University Hospital, n (%) | Total, N (%) |
| 15% or less | 5 (29) | 0 (0) | 0 (0) | 5 (13) |
| 16% to 25% | 2 (14) | 3 (22) | 1 (14) | 6 (17) |
| 26% to 35% | 4 (21) | 0 (0) | 1 (14) | 5 (13) |
| 36% to 45% | 5 (29) | 4 (33) | 3 (29) | 11 (30) |
| 46% to 55% | 1 (7) | 4 (33) | 4 (43) | 9 (23) |
| 66% to 75% | 0 (0) | 1 (11) | 0 (0) | 1 (3) |
| Total | 17 (100) | 12 (100) | 9 (100) | 38 (100) |
|  | | | | |

Table S3. Association between patients’ clinical prognosis and physicians’ action

| Clinical condition | Decision accepted without discussion, n (%) | Family counselled to investigate reason/convince decision maker to change decision, n (%) | Connect with social worker, n (%) |
| --- | --- | --- | --- |
| A child with a good prognosis whose caretakers refuse to start treatment |  | 7 (100) |  |
| A child with a poor prognosis whose caretakers refuse to start treatment | 2 (29) | 5 (71) |  |
| A child with a good prognosis undergoing treatment, whose caretakers refuse to continue treatment |  | 6 (86) | 1 (14) |
| A child with a poor prognosis undergoing treatment, whose caretakers refuse to continue treatment | 4 (57) | 3 (43) |  |
| Contact tracing practice | | | |
| During ongoing treatment, what will happen if a child misses a scheduled appointment for chemotherapy or radiotherapy or surgery | Child's family/caretaker would be contacted | It is not routine practice to contact the child's family/caretaker | Total |
|  | 2 (28) | 5 (72) | 7(100) |

Table S4. Pre-identified risk factors associated with treatment abandonment

| Variable | Extremely low likelihood, n (%) | Low likelihood, n (%) | No relation, n (%) | High likelihood, n (%) | Extremely high likelihood, n (%) | Total, N (%) |
| --- | --- | --- | --- | --- | --- | --- |
| Low economic status |  |  |  | 3 (8) | 35 (92) | 38 (100) |
| Cost of care |  |  |  | 4 (11) | 34 (89) | 38 (100) |
| Long travel time to treatment center |  |  | 1 (3) | 8 (21) | 29 (76) | 38 (100) |
| Belief in the incurability of cancer |  |  | 1 (3) | 11 (29) | 26 (68) | 38 (100) |
| Low level of parental education |  |  | 3 (8) | 10 (26) | 25 (66) | 38 (100) |
| Undernourishment of the child |  |  | 8 (22.8) | 16 (45.7) | 11 (31.4) | 38 (100) |
| Adverse effects and toxicity of treatment |  |  | 3 (8) | 22 (59.4) | 12 (32) | 38 (100) |
| Painful diagnostic and therapeutic procedures |  |  | 5 (14) | 24 (68.5) | 6 (17) | 38 (100) |
| Insufficient communication by healthcare professionals |  |  |  | 24 (63) | 14 (37) | 38 (100) |
| Preference for complementary and alternative medicine |  |  | 5 (13) | 23 (61) | 10 (26) | 38 (100) |
| Strong faith or religious beliefs |  |  |  | 29 (76) | 9 (24) | 38 (100) |
| HIV diagnosis of the child |  |  | 12 (35.3) | 15 (44) | 7 (20) | 38 (100) |
| Female sex |  |  | 37 (97) | 1 (3) |  | 38 (100) |
| Male sex |  |  | 37 (97) | 1 (3) |  | 38 (100) |
| Older age of the child or adolescence |  | 3(8) | 32 (89) | 1 (3) |  | 38 (100) |
| Younger age of the child |  |  | 26 (72) | 9 (25) | 1 (3) | 38 (100) |

Supplementary text S1. Level of influence of pre-identified factors

The healthcare providers were asked to indicate the level of influence of pre-identified risk factors on treatment abandonment at their treatment center. These risk factors were identified by the International Society of Pediatric Oncology Abandonment Technical Working Group.

We asked them to indicate the perceived likelihood of a risk factor leading to treatment abandonment by using the following options: strongly decrease likelihood, decrease likelihood, no relation, increase likelihood, strongly increase likelihood. At the analysis stage, we developed five categories (major role, important role, moderate role, minor role, and no relation) based on a combination of responses about the level of influence.

1. A factor was considered to play a major role in influencing abandonment if more than 85% of the respondents indicated that it had a “strongly increase likelihood” or “increase likelihood”, and provided that ≥65% of the respondents reported that it has a “strongly increase likelihood.” We assigned more value to risk factors labeled as having a “strongly increase likelihood.”
2. A factor was considered to play an important role if the cumulative reported frequency was greater than 65%, and if more than 40% of the respondents reported an “increase likelihood” or 25% to 65% of the respondents reported a “strongly increase likelihood.”
3. A factor was considered to play a moderate role if 40% to 65% of the respondents reported a strongly increase likelihood” or an “increase likelihood.”
4. A factor was considered to play a minor role if 20% to 40% of the respondents reported a “strongly increase likelihood” or an “increase likelihood.”
5. A factor was considered to not be related to abandonment if more than 80% of the respondents reported “no relation.”

Table S5. Availability of essential interventions for childhood cancer treatment in the included healthcare centers

| Interventions | Tikur Anbessa Specialized Hospital (n = 14) | | Gondar University Hospital (n = 9) | | Jimma University Hospital (n = 7) | | Total (n= 30) | |
| --- | --- | --- | --- | --- | --- | --- | --- | --- |
|  | available | not available | available | not available | available | not available | available | not available |
| Locally adopted treatment protocols | 47% | 53% | 58% | 42% | 86% | 14% | 53% | 47% |
| Effective procedural sedation and analgesia | 71% | 29% | 89% | 11% | 86% | 14% | 80% | 20% |
| Free chemotherapy | 14% | 86% | 0% | 100% | 86% | 14% | 27% | 73% |
| Subsidized chemotherapy | 100% | 0% | 17% | 83% | 100% | 0% | 77% | 23% |
| Free/subsidized surgery | 100% | 0% | 22% | 78% | 100% | 0% | 77% | 23% |
| Free/subsidized blood products | 100% | 0% | 100% | 0% | 100% | 0% | 100% | 0% |
| Financial support for travel | 57% | 43% | 11% | 89% | 100% | 0% | 53% | 47% |
| Free/subsidized food | 100% | 0% | 89% | 11% | 100% | 0% | 97% | 3% |
| Free/subsidized lodging | 93% | 7% | 0% | 100% | 100% | 0% | 67% | 33% |
| Social support | 100% | 0% | 89% | 11% | 100% | 0% | 97% | 3% |
